# Relative fat oxidation is higher in children than adults

**DOI:** 10.1186/1475-2891-6-19

**Published:** 2007-08-16

**Authors:** John C Kostyak, Penny Kris-Etherton, Deborah Bagshaw, James P DeLany, Peter A Farrell

**Affiliations:** 1 Department of Biology, University of Delaware, 305 Wolf Hall, Newark, Delaware, 9716, USA; 2 Department of Nutritional Sciences, The Pennsylvania State University, S-126 Henderson Bldg., University Park, Pennsylvania, 16802, USA; 3 Department of Medicine, University of Pittsburgh, BSTWR E1140, Pittsburgh, Pennsylvania, 15261, USA; 4 Department of Exercise & Sport Science, East Carolina University, 176 Minges Academic Wing, Greenville, North Carolina, 27858, USA

## Abstract

**Background:**

Prepubescent children may oxidize fatty acids more readily than adults. Therefore, dietary fat needs would be higher for children compared with adults. The dietary fat recommendations are higher for children 4 to 18 yrs (i.e., 25 to 35% of energy) compared with adults (i.e., 20 to 35% of energy). Despite this, many parents and children restrict dietary fat for health reasons.

**Methods:**

This study assessed whether rates of fat oxidation are similar between prepubescent children and adults. Ten children (8.7 ± 1.4 yr, 33 ± 13 kg mean ± SD) in Tanner stage 1 and 10 adults (41.6 ± 8 yr, 74 ± 13 kg) were fed a weight maintenance diet for three days to maintain body weight and to establish a consistent background for metabolic rate measurements (all foods provided). Metabolic rate was measured on three separate occasions before and immediately after breakfast and for 9 hrs using a hood system (twice) or a room calorimeter (once) where continuous metabolic measurements were taken.

**Results:**

During all three sessions whole body fat oxidation was higher in children (lower RQ) compared to adults (mean RQ= 0.84 ± .016 for children and 0.87 ± .02, for adults, p < 0.02). Although, total grams of fat oxidized was similar in children (62.7 ± 20 g/24 hrs) compared to adults (51.4 ± 19 g/24 hrs), the grams of fat oxidized relative to calorie expenditure was higher in children (0.047 ± .01 g/kcal, compared to adults (0.032 ± .01 p < 0.02). Females oxidized more fat relative to calorie expenditure than males of a similar age. A two way ANOVA showed no interaction between gender and age in terms of fax oxidation.

**Conclusion:**

These data suggest that fat oxidation relative to total calorie expenditure is higher in prepubescent children than in adults. Consistent with current dietary guidelines, a moderate fat diet is appropriate for children within the context of a diet that meets their energy and nutrient needs.

## Background

Progression through puberty includes rapid and major changes in many physiological processes. Several studies have demonstrated a decrease in insulin sensitivity during puberty [1,2]. Changes in insulin sensitivity or other factors such as the steroidal environment [3] could also alter fuel mobilization and utilization before and during puberty. Jones et al [4] found higher net fat oxidation in children (5–10 yrs), both post-absorptive and postprandial, than in adults. Unfortunately no other direct comparisons of fuel utilization, between pre-pubescent children and adults, seem to exist. If fuel metabolism is altered before or during puberty it is logical that macronutrient requirements also change during this period. However, additional data are needed to establish whether this occurs.

A very low fat diet that also is low in calories and does not meet energy needs will interrupt normal growth and development of young children [5-7]. Until recently, a diet that provides less than 30% of calories from fat was recommended for children over two years of age [8]. This guideline was translated by some in an overzealous, but well-intentioned, manner to provide as little fat as possible in the diet leading to inadequate energy intake and compromised growth. In 2002, the National Academies released "Dietary Reference Intakes for Energy and Macronutrients" for all age groups [9]. For children 1–3 years of age, the Acceptable Macronutrient Distribution Range (AMDR) for total fat is 30 to 40% of energy and 25 to 35% of energy for children 4 to 18 years of age. In adults, the AMDR for fat has been set at 20 to 35% of energy. Collectively, these total fat recommendations represent a "step-wise" reduction in fat intake. The 2005 Dietary Guidelines [10] for total fat are consistent with the AMDR for total fat. The impetus for this guidance was to assure adequate energy and nutrient intake. Demonstrating that relative (relative to calorie intake) fatty acid oxidation rates are higher in children vs. adults would provide additional affirmation for the new total fat guidelines for children. Consequently, we felt it was important to quantify whole body fatty acid oxidation rates in children versus adults, and determine whether they differed, and if so, by how much.

The measurement of whole body fat oxidation (under conditions where CO_2 _buffering is stable) is straightforward through the use of indirect calorimetry and, with proper scaling for body size, this method is valid for comparisons between children and adults. Identification and partitioning of sources of fatty acids entering beta-oxidation pathways are not as straightforward. One commonly used method is to trace infused or ingested radio or stable isotope labeled fatty acids either in the plasma and/or in breath. Heiling et al. [11] directly compared measures of fat oxidation derived from indirect calorimetry with that derived from ^14^C or ^3^H labeled FA and concluded that the two measures were not comparable. The physiological cause of this discrepancy may be that ingested or infused fatty acids are temporarily sequestered in intracellular pools which are not immediately reflected in either breath or plasma fatty acid specific activities. Because the report that compared fat oxidation in prepubescent children versus adults[4] depended heavily but not exclusively on stable isotopes, we felt it important to directly measure whole body fat oxidation using indirect calorimetry in children and adults. Studying both children and adults under the same experimental conditions permits comparisons to determine whether relative (i.e., on an energy expenditure basis) fat oxidation rates are higher in children than adults.

## Methods

### Subjects

Ten adults (5 men, 5 women) aged 27–55 yr and ten children (5 boys, 5 girls) aged 6–10 yr participated in this study after having a routine physical examination and screening for Tanner Stage status. All subjects were healthy and free from diseases known to alter resting metabolism (thyroid disease, diabetes, extreme under or overweight, retarded or excessive growth rates) and each had a body mass index within the non-obese range (less than 30 kg/m^2^). Thus excessively obese or lean children and adults were excluded from the study. Subjects were recruited from the general population by use of public advertisements in local newspapers and bulletin board postings. The study was approved by the Institutional Review Board of the Pennsylvania State University and informed written consent was obtained prior to subject involvement in any part of the study.

### Cardiovascular fitness test and body composition

Because regular endurance exercise training may enhance fat oxidation, subjects were chosen based on their living a normally active lifestyle and those participating in rigorous physical training programs were excluded. All subjects completed a maximal treadmill stress test that required walking until volitional fatigue. Heart rate, respiratory gases and ventilation were measured as previously described [12,13]. A leveling off of VO_2 _with increasing workload was used as the criteria for reaching maximal oxygen consumption (VO_2_max). However, three of the children stopped the test before meeting this criteria. Their VO_2_max values were estimated by linear regression (VO2- HR) analysis and use of the age predicted maximal heart rate. Cardiovascular fitness testing was performed because regular exercise training may result in a greater utilization of fat which could complicate the interpretation of the results although studies exist which both support [14] and refute [15] this concept. Body fat was measured by skin-fold test with the same trained technician making all measurements on all subjects. The accuracy of his assessments was validated against results obtained from a separate groups of subjects (n = 16) using underwater weighing as the criterion measure[16,17]. Seven skinfold sites were measured and constants and weighting factors used for children 6–11 yrs were those suggested by Lohman [18].

### Diet

The 3-day diet cycle was designed to be child friendly, convenient and easy to prepare using the NUTRITIONIST V database (N-Squared Computing, First Data Bank Division, San Bruno, CA). Subjects were fed the prepared diet for three continuous days and on the fourth day a metabolic assessment occurred. The identical three-day diet was repeated for each of the three experimental sessions (room calorimeter and two days using a metabolic hood system) as described below. Caloric needs were calculated to maintain current body weight using the Harris-Benedict Equation with an activity factor of 1.5. The diet macronutrient targets were: 13–18% protein, 50–55% carbohydrates, and 30–35% fat. All three menus met these requirements. Macronutrient information is provided in Table 1. This experimental diet is representative of a typical American diet based on NHANES dietary data. For example, the average intake of SFA, MUFA, and PUFA are: 11%, 15%, and 7%, respectively. In addition, fiber intake is consistent with current average intakes of adults and children in the U.S. Furthermore, dietary cholesterol in the experimental diet is representative of cholesterol intake in adult males, females and children [19,20].

**Table 1:** Diet Analysis (example for ~2000 kcal menu) *

	Day 1	Day 2	Day 3	3- Day Average
Kcal	1987	1972	2008	1989
Carbohydrates (% en)	51	53	50	51
Protein (% en)	16	13	16	15
Total Fat (% en)	35	35	36	34
SFA (% en)	14	13	12	13
MUFA (% en)	13	12	9	11
PUFA (% en)	3	7	7	6
Cholesterol (mg)	163	264	100	176
Fiber (g)	20	12	22	18

* analysis from Nutr V database

Subjects were provided all food for three days prior to the test day and were instructed to consume only foods provided. Portion sizes were adjusted on the basis of each subject's estimated calorie requirements. Beverages were included with the prepared foods (milk and juice) and participants were instructed that they could drink other non-caloric, non-caffeinated beverages as desired. All food was weighed to the nearest tenth of a gram, and packed in a cooler for take out, to be eaten at home. This method of feeding was successful in that no significant weight gain or loss occurred for each individual from the first to the last experiment.

### Protocol

Prior to testing, subjects were familiarized with metabolic testing in both a room calorimeter and a flow-through hood system. Once adapted to the testing environment, each subject's experimental sessions were scheduled at least one week apart (range = 1–3 wks). In a random order, subjects completed metabolic measurements using a hood calorimeter on two occasions and they lived in a room calorimeter for the other session. Upon arrival at the laboratory the subjects voided and were weighed. Subjects were fasted overnight prior to all metabolic testing. Subjects rested supine for 30 minutes under a ventilated clear plexiglass hood every hour for eight hours. On another day subjects stayed for 9 hours in a room calorimeter. Both systems, hood and room calorimeter, used Hartmann Braun oxygen (Magnos 4G) and carbon dioxide (Uras 4) analyzers. Calibration gases were analyzed using both mass spectrometry (supplier analysis) and the Scholander micro technique [21] in our laboratory. The room calorimeter was tested using several controlled alcohol burns to measure CO_2_. This calibration revealed that CO_2 _production could be measured with a recovery accuracy of >96%. Analyzers were calibrated immediately before use in the morning, periodically throughout the day (for the first few subjects, until we became confident in the consistency of the analyzers over the course of the experiment) and immediately after the last gas collection.

Ensure^®^, a supplement meal replacement drink, was provided as a standard means to deliver all meals of known macronutrient composition during metabolic measurements. The meal was heated to a desired temperature (85°F) [22]. Ensure contains 14.1% of calories from protein, 22% of calories from fat (1.9% saturated, 6.7% polyunsaturated, 11.8% monounsaturated) and 63.9% of calories from carbohydrate. Both children and adults consumed the Ensure without incident and this was the only calorie containing beverage or food allowed during the experiment. Subjects were given 15 minutes to consume all of the Ensure. Each meal comprised 30% of the subject's estimated daily caloric needs (RMR × 1.2 for activity levels). This activity constant was reduced from the value usually assigned for free living conditions because during the experimental day subjects were instructed to maintain low levels of spontaneous physical activity in the laboratory.

For the hood measurement day, subjects rested in bed for 20 minutes in the semi-recumbent position prior to the first metabolic measurement. Upon completion of this first metabolic measurement subjects ingested breakfast as described above. During the room calorimeter experiments, the subjects entered the room after voiding and were served breakfast (Ensure) 15 minutes later. The reason for the slight difference in protocols between room and hood experiments was that it takes about 25 minutes for the room calorimeter to reach equilibrium after the doors are sealed. Subjects were inactive for the duration of the tests and were supervised constantly. No planned or spontaneous physical exercise was allowed. Children and adults watched movies, read books or played games. Physical exertion was limited to slow walking in the building. During each test day, only bottled water was permitted in addition to the test meal. Lunch was identical to breakfast and was served 4 hrs after breakfast. All urine produced during each test day was collected, pooled, had the volume measured and a small sample was assayed for urea nitrogen.

Heart rate (HR) and blood pressure (BP) were monitored every other hour during the hood collections days and before and after the room calorimeter days. The child's size blood pressure cuff of the Dynamap instrument (HR and BP) was used for children. Subjects were weighed (after emptying the bladder and in the same clothes) to the nearest 100 g before and after the experiments.

### Calculations and Statistical Analyses

Total fat grams oxidized were calculated from the equation, g fat/min = 1.67 VO_2 _- 1.67 VCO_2 _- 1.92 N, where VO_2 _and VCO_2 _are in L/min and N is the rate of excretion of urinary UREA nitrogen in g/min[23]. The grams of fat oxidized using this equation were expressed for the total collection period. We realize that this extrapolation probably underestimates values for fat oxidation which would be found under free living conditions where activity is not restricted. Statistical analysis included tests for normalcy of the data and then parametric tests such as paired t-test for two group comparisons (children vs adults) and ANOVA for four group comparisons (boys, girls, men, women). When significant F ratios were found through ANOVA, a Student- Newman- Keuls multiple comparison post hoc test was used to locate means which differed significantly. To determine whether an interaction occurred between gender and age in terms of mean differences in rates of fat oxidation, a 2 factor ANOVA was conducted. A 0.05 level of confidence was chosen a priori as being significant.

## Results

Subject characteristics are presented in Table 2. Body weight of the adults and children remained stable, 0.25 ± 0.26 and 0.13 ± 0.13 kg, respectively, for the room calorimeter day and similar non-significant changes occurred on the hood days. Values for VO_2 _and VCO_2 _obtained for the two hood experiment days were statistically identical as were average heart rate and blood pressure recordings for each group. Heart rate averaged between 80–85 bpm (range)for children and between 62–74 bpm for adults on both hood days. Mean arterial pressures also were within normal ranges with no marked elevations or declines during the testing period.

**Table 2:** Characteristics of Subjects by Group

	Age (yrs)	Height (cm)	Weight (kg)	% Body Fat	BMI (kg/m^2^)	VO2 Max (ml/kg/min)	Urinary N_2 _(total g/24 hr)
Adults (n = 10)	41.6 ± 8.5	170.6 ± 10.9	73.6 ± 13.2	27.08 ± 5.43	25.1 ± 2.8	36.7 ± 10.7	12.7 ± 3.5
Children (n = 10)	8.7 ± 1.4	136.4 ± 13.6	33.9 ± 13.9	17.35 ± 11.09	17.7 ± 3.9	42.7 ± 9.2	7.5 ± 2.4

BMI: body mass index

**Figure 1:**
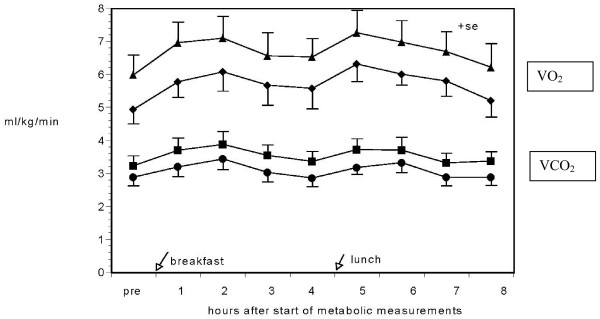
Oxygen consumption and carbon dioxide production for each group during the background hood day. Symbols (Triangle = child VO_2_, Diamond = child VCO_2_, Square = adult VO_2_, Circle = adult VCO_2_). N = 10 per group. Values are means ± SE.

The VO_2 _and VCO_2 _data show similar patterns for both the children and the adults, Figure 1. However, as expected, children had significantly, p < 0.05, higher values for both VO_2 _and VCO_2 _when expressed relative to body weight (ml/kg. min^-1^). Calculated kcals expended on a 24 hr basis were within expected ranges, taking into account the restricted physical activity. Men showed the highest mean value at 2072, then boys 1496, women at 1329 and finally girls at 1194 kcals/day. When energy expenditure was normalized to lean body mass (LBM), no significant difference was found between men (29.7 ± 1.49) and women (29.48 ± 2.7 kcal/d/kg LBM). However, values for children were higher (50.4 ± 6.9 for boys and 52.4 ± 5.3 for girls), p < 0.05 compared to adults.

**Figure 2:**
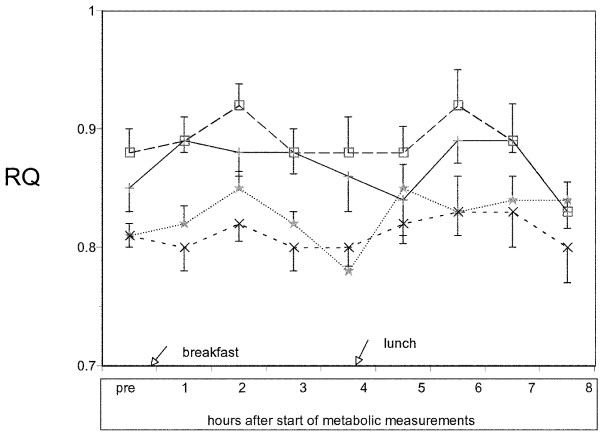
RQ values for each of the groups on the hood day. (□ = Adult male, n = 5), (+ = Adult female, n = 5), (* = Children male, n = 5), (x = Children female, n = 5). Values are means ± SEM.

**Figure 3:**
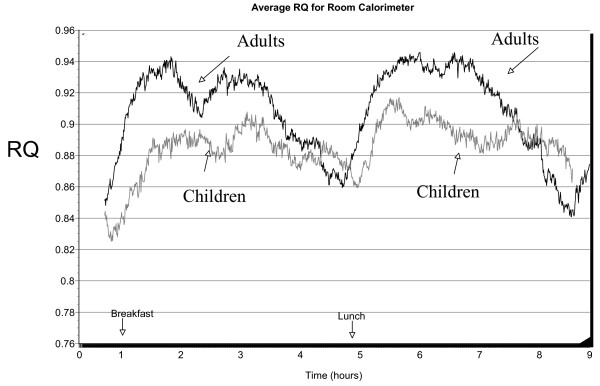
Average RQ values for children (n = 10) and adults (n = 10) for the room calorimeter day. For graphical clarity, indicators of variability around the mean are not presented. Each line represents a tracing of the average value for all ten subjects in each age group.

A consistent age effect for RQ was observed on all collection days (Figures 2 and 3). Using only the data collected for the hood measurement days (combined because there was no statistical difference between days), we calculated the total grams of fat oxidized for each sub-group for 9 hours using standard equations that included correction for urea nitrogen excretion. Total grams of fat oxidized were somewhat but not significantly higher in children (23.8 ± 7.6 g/9 hrs) compared to adults (19.28 ± 7.2 g/9 hrs). In an attempt to determine the contribution of fat oxidation to daily calorie expenditure, we calculated the grams of fat oxidized per kcal of energy expenditure. This value was higher in children (0.047 ± 0.01 g/kcal) compared to adults (0.032 ± 0.01, p < 0.02). When groups were analyzed according to gender and age, a 2 factor ANOVA showed fat oxidation for females, both children(.052 ± .016 g/kcal) and adults(.042 ± .014) were significantly higher (F = 6.07, p = .025) than values obtained for males of a comparable age (boys .041 ± .006 and men .023 ± .013 g/kcal). Children, both girls and boys, had higher fat oxidation rates relative to adults (F = 5.6, p = .03). The F ratio for gender by age interactions was not significant, (F = 0.54, p = .47).

## Discussion

The results of this study, based on indirect calorimetry on three separate days, suggest that pre-pubertal children have a higher relative rate of whole body fat oxidation than adults. By the term relative we mean that the number of grams of fat that are oxidized in a certain time frame divided by the total calorie expenditure for that time frame. The absolute amount (g/hr) of fat oxidized by adults was not statistically different between adults and children probably due to age dependent differences in body size and LBM in children and adults. Prepubescent children may oxidize more fat relative to total energy expenditure than adults for the purpose of supporting normal growth processes such as higher rates of protein synthesis, lipid storage and bone growth.

The average RQ values shown in Figures 2 and 3 are within the range found in normal weight children by Riddell[24] (10–14 yr) and McCann[25] (6–13 yr) and are slightly lower than those reported by Molnar et al. [26] (9–16 yr), Maffeis et al. [27], and Goran and Nagy[28] (4–9 yr). Postprandial RMR values found in this study are similar to those of Ventham and Reilly[29] (using laboratory measures) as well as those of free-living children of similar age using ^2^H_2 _^18^O methodology[30,31]. In addition, the calculated grams of fat oxidized per day in our study is almost identical to that reported by Jones et al. [4] and Salbe et al. [32] and only slightly higher than those reported by Maffeis et al. [27]. Thus, the RQ and RMR data are quantitatively similar to those reported for children of similar age in the literature.

It is possible that there were differences in the psychological response to the test protocols between adults and children that accounted for the different fat oxidation rates. Heightened arousal could alter fuel selectivity; however, we are not aware of studies in this area. We were especially cognizant of the fact that isolation while in the room calorimeter might be more stressful for children. Both adults and children spent time in the room calorimeter on a familiarization day prior to actual testing. The children involved in this study tolerated the time in the calorimeter room very well by engaging in quiet play, games and other relaxing activities. The door of the calorimeter has a window so that subjects can interact with experimenters, if needed, and one wall of the calorimeter had a window view to the outside. While not quantified, our subjective impression was that children were comfortable in the room calorimeter in a manner similar to the adults.

The number of subjects in each group is small and this limits the ability to generalize to the overall population. However, we made three independent and repeated measures of this difference using two different measurement systems (hood and room). With such a small number of subjects it is possible that gender could influence our findings. Adult males oxidized significantly less fat relative to metabolic body size than young males and adult females also oxidized less fat than young females however, that difference was not statistically significant. We speculate that due to earlier maturation in females, metabolic pathways in the girls resembled those of women more closely than those found between men and boys. We suggest that an investigation using a greater number of subjects be performed to verify our results. It would also be valuable to know whether ethnicity influences our findings.

The majority of data collected and presented in this study represent non-resting conditions however differences in resting metabolic rate, i.e. the starting metabolic rate before a perturbation, could influence the interpretation of our results. We calculated resting metabolic rate using that first measurement of the day and our results are quite similar to those provided by Poehlman and Toth [30]. Resting metabolic rate averaged 4.93 ± 1.09 for adults and 3.91 ± 0.98 kJ/min for children. Differences in body size require caution in interpreting resting energy expenditure when children and adults are compared. As an example, when fat free mass (x axis, kg) is regressed against resting metabolic rate (RMR, kJ/min), the y intercept is not zero. This non zero intercept can lead to a statistical bias when comparing data from groups that differ greatly in metabolic body size. Our data for adults in this respect are similar to those of Poehlman and Toth[30] who found Y intercepts of 1.35 and 1.51 for women and men respectively. Our intercept for adults was 1.3. One way to eliminate this bias is to use analysis of covariance with LBM as the co-variate which removes the effect of LBM on RMR. When we preformed this analysis the F ratio (F = 7.7, p = .08) showing that the differences between adults and children for RMR was not significant. Thus the greater rate of fat oxidation in children is not due to a greater metabolic rate. It should be emphasized, however, that the only true resting measurements taken in this study were the first measurement of the day before the standard breakfast that was provided and our intention in this work was not to determine whether resting metabolic rates were similar between children and adults. Within the limitations of our experimental conditions, the present study has shown that children oxidize greater amounts of fat per calorie expended each day compared to adults. This is supportive of a higher total fat dietary recommendation for children versus adults. However, this interpretation needs to be framed in the context of energy balance and nutrient adequacy. This is particularly important given the increasing incidence of overweight/obesity in children in the U.S. [33]. Nonetheless, as is apparent, sufficient fat must be included in the diet for children to support normal growth and development. Because there is a recommended fat range for children of different ages it is important to provide flexibility in diet planning, and to facilitate adherence to the fat recommendations that have been made.

## Competing interests

The author(s) declare that they have no competing interests.

## Authors' contributions

JCK conducted all data collection using the room calorimeter and hood systems, recruited subjects, analyzed the data and wrote the manuscript. PKE helped design the experiments, obtain IRB approval, helped write the manuscript, coordinated meal development. DB supervised all aspects of meal preparation and delivery. JPD helped design the experiment, sample analysis, data analysis, writing the manuscript and interpretation of results. PAF helped design the experiment, obtain IRB approval, collect data, recruit subjects and write the manuscript.
